# Managing a functional disorder with vertigo or dizziness in a primary care setting: Clinical case

**DOI:** 10.1192/j.eurpsy.2023.1435

**Published:** 2023-07-19

**Authors:** K. Tzartzas, S. Aslan, I. Kokkinakis

**Affiliations:** Département des Policliniques (DDP), Unisanté - Centre de médecine générale et de santé publique, Lausanne, Switzerland

## Abstract

**Introduction:**

A heterogeneity in prevalence rates of functional and/or dissociative disorders is evidenced in primary care settings. At least one medically unexplained symptom is diagnosed in 40–49% of all primary care patients and 91% of all patients with a functional diagnosis are managed exclusively by general practitioners (GP) and nonpsychiatric specialists. It is therefore important that GPs identify these disorders so that individualized, multimodal treatment, with psychiatric collaboration, can be initiated promptly.

**Objectives:**

Our objective is to demonstrate the role of consultation-liaison (CL) psychiatrists in the management of patients with a functional diagnosis in primary care, as well as the potential impact of non-collaboration between GPs and psychiatrists.

**Methods:**

Case report of Mrs. P., a 32-year-old patient, married for one year. Following a burnout that occurred two years ago, associated with anxiety-depressive symptoms, she developed gradually persistent dizziness, with balance disorders and asthenia. Mrs. P. consulted a psychiatrist for these symptoms and was treated first with an SSRI and then with an SNRI, which increased her symptoms of dizziness and vertigo. She stopped the psychiatric treatment, being disappointed by the proposed care, and asked her GP for help. No pathology was revealed by the neurological and ENT assessment requested by her GP. He referred her for a second opinion at the university center for general medicine.

**Results:**

After an initial GP assessment, a CL-psychiatric evaluation was performed (a first joint GP-psychiatrist session, 3 psychiatric sessions, and a feedback joint GP-psychiatrist session), during which a feeling of loss of control was noted in a patient with obsessive personality traits and controlling tendencies. A bidirectional relationship between anxieties, underlying uncontrolled internal conflicts, and dizziness was demonstrated. A dynamic work around the underlying conflicts according to the bio-psycho-social model allowed to identify the presence of a dissociative neurological symptom disorder, with vertigo or dizziness (6B60.2) of which the patient could become aware. This brief CL-psychiatric and psychotherapeutic intervention, proposed and accompanied by the GP, made it possible to explore and elaborate on the patient’s modes of functioning in her relationship to her body, to herself, and to others. At the same time, vestibular rehabilitation was performed by a ENT, with a favorable clinical and postural evolution. Thanks to this multidisciplinary treatment led by the GP, Ms P. was able to resume her professional and social activities after 3 months.

**Conclusions:**

GPs have a central role in the detection of dissociative neurological symptom disorder, with vertigo or dizziness, and in the rapid organization of an adapted care network. Collaboration with CL-psychiatrist can offer optimal management of such disorders in primary care settings.

**Disclosure of Interest:**

None Declared

